# Inhibitory Activity of Compounds Obtained from *Streptomyces* Against *Trypanosoma cruzi*

**DOI:** 10.3390/pathogens14070638

**Published:** 2025-06-26

**Authors:** Jorge Andrés Delgado-Garduño, Lucio Galaviz-Silva, Ma Guadalupe Rojas-Verde, Joel Horacio Elizondo-Luevano, Lidia Baylón-Pacheco, José Luis Rosales-Encina, Guadalupe Gutiérrez-Soto, Zinnia Judith Molina-Garza

**Affiliations:** 1 Universidad Autónoma de Nuevo León, Facultad de Ciencias Biológicas, Laboratorio de Patología Molecular y Experimental, Ciudad Universitaria, Ave. Universidad, S/N, San Nicolas de los Garza 66455, Nuevo León, Mexico; jorge.delgadogrd@uanl.edu.mx; 2 Universidad Autónoma de Nuevo León, Facultad de Ciencias Biológicas, Instituto de Biotecnología, Laboratorio 8, Ciudad Universitaria, Ave. Universidad, S/N, San Nicolas de los Garza 66455, Nuevo León, Mexico; guadalupe.rojasvrd@uanl.edu.mx; 3 Instituto de Investigación Biomédica de Salamanca, Facultad de Farmacia, Universidad de Salamanca, Campus Miguel de Unamuno S/N, 37007 Salamanca, Spain; jelizondo@usal.es; 4 Universidad Autónoma de Nuevo León, Facultad de Agronomía, Biomolecular Innovation Group, Laboratorio de Ciencias Naturales, Campus Ciencias Agropecuarias, Francisco I. Madero S/N, Ex Hacienda el Canadá, Cd. Gral. Escobedo 66050, Nuevo León, México; juanita.gutierrezst@uanl.edu.mx; 5 Departamento de Infectómica y Patogénesis Molecular, Centro de Investigación y de Estudios Avanzados de IPN. Av. Instituto Politécnico Nacional No. 2508, Col. San Pedro Zacatenco, Del. Gustavo A. Madero, Ciudad de México 07360, Mexico; lbaylon@cinvestav.mx (L.B.-P.); rosales@cinvestav.mx (J.L.R.-E.)

**Keywords:** *Streptomyces thermocarboxydus*, Chagas disease, *Artemia salina*, aglycone staurosporine, amphomycin, antiparasitic activity

## Abstract

Chagas disease (ChD) caused by *Trypanosoma cruzi* remains a major public health concern, affecting approximately 8 million people worldwide. However, the number of undiagnosed cases is likely much higher. Existing treatments rely on benznidazole and nifurtimox which, despite their efficacy during the acute phase of infection, are often associated with severe side effects that can be life-threatening. As a promising alternative, actinomycetes—which are renowned for producing pharmacologically and industrially relevant metabolites—have demonstrated potent antimicrobial properties; however, their antiparasitic potential remains largely unexplored. This study evaluated the anti-trypanocidal activities of extracellular metabolites produced by *Streptomyces thermocarboxydus* strain Chi-43 (ST-C43) and *Streptomyces* sp. strain Chi-104 (S-C104) against epimastigote, trypomastigote, and amastigote forms of *T. cruzi*. The strains were cultured in ISP2 broth, and their extracellular metabolites were assessed via antiparasitic diffusion assays in microplates. The 50% lethal concentration (LC_50_) values ranged from 102 to 116 μg/mL against epimastigotes and trypomastigotes. The antiparasitic activity was confirmed through 3-(4,5-dimetiazol-2-yl)-2,5-diphenyltetrazolium bromide (MTT)-based spectrophotometric assays and optical microscopy. Toxicity assays revealed that the extracellular metabolites were non-toxic to *Artemia salina*, non-cytotoxic to Huvecs, and non-hemolytic to human erythrocytes. Dose–response regression analysis showed statistically significant differences (*p* ≤ 0.05). LC-MS/MS analysis identified amphomycin and K-252c aglycone staurosporine as the active antiparasitic compounds. These findings highlight the potential of *Streptomyces*-derived extracellular metabolites as novel, selective, and safe anti-*T. cruzi* agents. Nevertheless, further studies in murine or preclinical models are needed to validate their efficacy and support future clinical applications for the treatment of ChD.

## 1. Introduction

Chagas disease (ChD), also known as American trypanosomiasis, is caused by the protozoan *Trypanosoma cruzi* (Chagas, 1909). This neglected tropical disease is endemic to Mexico, Central America, and South America, and is often referred to as a “silent” illness because many individuals remain unaware of their infection. It is estimated that over 7 million people are currently infected worldwide, with approximately ~40,000 new cases and more than 10,000 deaths reported each year [1,2]. In Mexico, around 1.1 million people have been infected by this hemoflagellate parasite [3].

Although ChD predominantly affects rural populations, urban areas have also seen a rise in cases, largely due to people migrating from impoverished rural regions to major cities in search of employment opportunities [4]. The protozoan hemoflagellate is transmitted primarily through the feces of triatomine insects containing metacyclic trypomastigotes. However, other modes of transmission include blood transfusions, organ transplants from infected donors, congenital transmission (from mother to fetus), oral ingestion of contaminated food or beverages, and accidental exposure in laboratory settings [5].

Once acquired, the infection progresses through two clinically distinct stages—an acute phase and a chronic phase—both of which are often misdiagnosed. The acute phase may be asymptomatic or present with non-specific symptoms, although severe manifestations can also occur [6]. This is followed by an indeterminate asymptomatic period, which can last for years. Eventually, some individuals progress to the chronic phase, which may remain asymptomatic or develop into serious clinical conditions, including cardiomyopathy and gastrointestinal complications involving the esophagus, intestine, or colon [7].

Two drugs are primarily used for the treatment of ChD: benznidazole and nifurtimox. These are most effective when administered during the acute phase of the infection [8]. The goal of treatment is to prevent potential organ damage and reduce the risk of parasite transmission [9]. The recovery rate with these drugs is approximately 70% [10]. However, their use is typically accompanied by significant disadvantages, including the development of adverse reactions such as skin rashes, neuropathy, nausea, vomiting, and diarrhea, in addition to their limited efficacy during the chronic phase. Consequently, there is a pressing need to explore more effective therapeutic alternatives that are free from these side effects.

Given the limitations of existing treatments and the growing global burden of ChD, the search for new therapeutic agents is critical. Natural products from plants and microorganisms have demonstrated antitrypanosomal activity with non-cytotoxic activity against certain cell lines, including extracts and pure compounds derived from *Terminalia* plants which range from moderately to highly active; for example, ellagic acid, punicalagin, and flavogallonic acid [11]. Strasseriolides isolated from the fungus *Strasseria geniculate* have also been shown to possess activity against extracellular trypomastigote and intracellular amastigote forms of *T. cruzi,* which is particularly desirable [12]. Additionally, a neolignan compound isolated from the flowers of *Nectandra leucantha* exhibited potent activity against the intracellular forms of *T. cruzi* (amastigotes), with an LC_50_ (half-maximal inhibitory concentration) value of 4.3 μM [13]. Furthermore, some microorganisms—such as the marine obligate bacterium *Microbulbifer*—yield a cyclic hexapeptide, called bulbiferamide, which presented growth inhibitory activity with an LC_50_ value of 4.1 μM, comparable to that of the approved drug benznidazole [14].

Actinomycetes, which are known for their ability to produce bioactive secondary metabolites, represent a promising source of novel antiparasitic compounds, and may offer more effective and safer alternatives to traditional treatments [15]; for example, phenazine-1-carboxylic acid (PCA) showed anti-protozoal activity against *Toxoplasma gondii* (LC_50_: 55.5 μg/mL) and *Plasmodium falciparum.* In particular, this compound was identified in the active metabolite fractions derived from *Streptomyces canus* [16]. Venturicidin A, a macrolide, has been demonstrated to be highly active against *Trypanosoma brucei* and *Leishmania donovani* [17]. Recently, the natural anti-kinetoplastid activities of indolocarbazole staurosporine (mainly 7-oxostaurosporine) have been confirmed, which was isolated from cultures of *S. sanyensis* and shown to be active against *Acantamoeba castellanii, L. amazonensis; L. donovani, and T. cruzi* [18].

Actinomycetes have long been recognized as a valuable source of antibiotics and are known to produce a wide range of bioactive compounds with antimicrobial properties [15]. Despite this, the full potential of these compounds remains underexplored in the field of clinical parasitology, particularly in the context of *T. cruzi* infection [19,20]. This study aimed to assess the anti-*T. cruzi* activity of extracellular metabolites from *Streptomyces sp.* Chi-104 (S-C104) and *Streptomyces thermocarboxydus* Chi-43 (ST-C43) against a *T. cruzi* strain isolated from Nuevo León, Mexico, in order to evaluate their therapeutic potential for ChD.

## 2. Materials and Methods

### 2.1. Ethical Statement

Experiments using human erythrocytes were conducted with the informed consent of a healthy donor in compliance with the Official Mexican Technical Standard for the Disposal of Human Blood and its Components for Therapeutic Purposes [21].

### 2.2. Streptomyces

The *Streptomyces* strains S-C104 and ST-C43 were isolated by M.G.R.-V. from the soil of Arareco Lake in Chihuahua, Mexico (27°42′42″ N, 107°35′31″ W) in April 2015.

### 2.3. Activation of Streptomycetes

The selection of the actinomycete strains was based on their superior hydrolytic activity. To reactivate the strains stored at −20 °C, they were cultured under sterile conditions on oat agar medium (Sigma Aldrich, Burlington, MA, USA), which contained magnesium sulfate (1 g), monopotassium phosphate (1 g), sodium nitrate (1.5 g), oats (1 g), and bacteriological agar (18 g), all dissolved in 1 liter of distilled water at a pH of 7.8. A 5 µL aliquot of each strain was inoculated onto sterile oat agar Petri dishes and incubated at 30 °C for 5–7 days [22].

### 2.4. Culture of Trypanosoma cruzi Epimastigotes

A culture of *T. cruzi* epimastigotes—isolated from *Triatoma gerstaeckeri* collected in General Terán, Nuevo Leon, Mexico in 2007 and belonging to discrete typing unit (DTU) 1 [23]—was prepared with Liver Infusion Tryptose (LIT) medium (Thermo Fisher Scientific, Waltham, MA, USA), supplemented with 20 μM hemin (Sigma Aldrich) and 10% (*v/v*) fetal bovine serum (FBS; Gibco, Grand Island, NY, USA), and incubated until exponential phase at a concentration of 1 × 10^6^ cells/mL was reached. The number of cells was confirmed via counting in a Neubauer chamber, and is expressed as number of parasites per milliliter (mL) of culture medium (parasites/mL) [24].

### 2.5. Antagonistic Activity Assay of Streptomyces Against Trypanosoma cruzi

Concentrations of 1 × 10^7^–5 × 10^7^ cells/mL of streptomyces and 1.2 × 10^6^ cells/mL of *T. cruzi* were prepared using the abovementioned cultures (Section 2.3 and Section 2.4), and then transferred into a 24-well microtiter plate and incubated at 28 °C for 24 h. Afterwards, the motile parasites were counted with a Neubauer chamber under an optical microscope (DM750 Leica, Wetzlar, Germany), in order to verify the inhibition due to exposure to the *Streptomyces* strains. The percentage of inhibition was calculated as previously reported [25].

### 2.6. Extraction of Compounds

Actinomycete biomass was inoculated in 250 mL of International *Streptomyces* Project-2 Medium broth (Thermo Fisher Scientific; ISP2: yeast extract 4 g, malt extract 10 g, dextrose 4 g, dissolved in 1 L of bidistilled water) [26] and incubated at 150 rpm and 28 °C for 15 days. The cultures were filtered with Whatman No. 1 paper 0.22 µm (Sigma-Aldrich). The active compounds were extracted with 1:1 ethyl acetate/actinomyces culture [27] at 50 rpm for 24 h. The organic phase was separated with a funnel, and the extracellular metabolites were concentrated under vacuum with a rotary evaporator (Yamato Digital, RE301, Santa Clara, CA, USA) at 40 °C. Finally, the yield percentage was calculated based on the dry weight obtained from the extracellular metabolites after incubation [28,29].

### 2.7. Antiparasitic Activity of the Extracellular Metabolite Extracts Against Epimastigote, Trypomastigote, and Amastigote Forms of Trypanosoma cruzi

To assess the anti-*T. cruzi* activity, 190 µL of LIT medium (Thermo Fisher Scientific) containing 1.2 × 10^6^ epimastigotes/mL and 10 µL of actinomycete extracellular metabolites (diluted in 1% dimethyl sulfoxide, DMSO) (Sigma-Aldrich) at concentrations ranging from 50 to 300 µg/mL were added to a 96-well microplate (Corning Incorporated, Corning, NY, USA) [30]. LIT medium with the extracellular metabolites served as the blank control, while 10 µg/mL nifurtimox (Sigma-Aldrich, St. Louis, MO, USA) was used as the positive control and 1% DMSO (at a volume equal to the extracellular metabolites) with LIT medium served as the negative control. All assays were performed in triplicate. The plates were incubated at 28 °C for 72 h [31,32].

Epimastigote viability was assessed using an enzymatic micro-method with MTT (3-(4,5-dimethylthiazol-2-yl)-2,5-diphenyltetrazolium bromide, Affymetrix, Cleveland, OH, USA), with optical density measured at 570 nm using a microplate reader (Biochrom Asys UVM-340, Cambridge, UK) at 27 °C for 4 h [26]. Subsequently, 100 µL of 10% sodium dodecyl sulfate (SDS)-0.1 N HCl solution (Sigma-Aldrich, Burlington, MA, USA) was added to extracellular metabolites with formazan crystals, and the plate was incubated for an additional 4 h. The mortality percentage of epimastigotes was calculated as previously described [31].

Regarding the count in the Neubauer chamber, 20 µL of the extracellular metabolites/parasite stock solution was used to determine the count of living epimastigotes. The results are expressed as the lethal concentration 50 (LC_50_), as previously reported [33].

Antiparasitic activity tests of the extracellular metabolites against trypomastigote and amastigote forms were carried out in the same way as for the epimastigotes, but with incubation periods at 28 °C for 24 h. 

### 2.8. Evaluation of the Toxic Activity of the Extracellular Metabolites in Artemia Salina Model

This test was carried out with *Artemia salina* (brine shrimp, INVE, Aquaculture, NV, USA), as a preliminary method to measure toxicity [34]. First, brine shrimp eggs were incubated in 3.7% sterilized artificial seawater (Kent, Frankli, WI, USA) in a disinfected glass aquarium with a pH of 8 and 25–30 °C. Aeration was provided using an aquarium pump, and the aquarium was exposed to constant light. Then, seawater was supplemented with 0.75 g/L yeast extract (BD Bioxon, Becton, Dickinson, NJ, USA). After 48 h of hatching, the bioassay was carried out in a 24-well plate, for which 10 larvae were transferred to each well. The extracellular metabolites were evaluated in triplicate at concentrations of 50–300 μg/mL. Potassium dichromate (K_2_Cr_2_O_7_, Sigma-Aldrich) at 100 μg/mL was used as the positive control, and 3.7% seawater was used as the negative control. The microplates were incubated at room temperature for 24 h under constant illumination. After exposure, the numbers of dead and alive nauplii were counted. To consider the bioassay valid, the percentage of mortality of the negative control should not exceed 10% [35].

### 2.9. Evaluation of Hemolytic Activity of the Extracellular Metabolites

Human blood samples were obtained from a healthy volunteer and collected in EDTA anticoagulant tubes (BD Vacutainer, Becton-Dickinson, Franklin Lakes, NJ, USA). Red blood cells were washed three times with sterile phosphate-buffered saline (PBS), pH 7.2. A 5% suspension of erythrocytes in sterile PBS was prepared from the last wash. Concentrations of 50–300 μg/mL of each strain’s extracellular metabolites were added in triplicate to a PBS solution, to which a 5% suspension of human erythrocytes was added, followed by incubation at 37 °C for 30 min. Then, the cells were centrifuged at 3000 rpm (Sorvall Legen Micro 21R, Thermo Fisher Scientific) and the supernatant was used to measure the absorbance of released hemoglobin at 540 nm. For the positive and negative controls, distilled water and PBS were used, respectively [36,37]. Extracellular metabolites that presented a hemolysis percentage of less than 10% at 1000 μg/mL were considered non-hemolytic [38,39].

### 2.10. Production of Trypanosoma cruzi Trypomastigotes

Trypomastigote forms of *T. cruzi* were obtained from a 10 mL culture of epimastigotes (1.2 × 10^6^ parasites/mL) harvested at the end of the exponential growth phase. The culture was centrifuged at 10,000× *g* for 15 min at 10 °C, and the resulting pellet was resuspended in artificial triatomine urine (TAU: 190 mM NaCl, 8 mM PBS, pH 6.0, 17 mM KCl, 2 mM CaCl_2_, 2 mM MgCl_2_). The suspension was incubated at 28 °C for 2 h to induce differentiation into trypomastigote forms. Subsequently, the parasites were diluted in TAU culture supplemented with 10 mM L-proline (TAUP) and incubated at 27 °C to promote further development [40,41,42].

### 2.11. Production of Trypanosoma cruzi Amastigotes

The induction of amastigotes of *T. cruzi* was performed with 3 × 10^6^ cells/mL of cultured epimastigotes, which were added to Human Umbilical Vein Endothelial Cells (Huvecs) (Sigma-Aldrich) in RPMI medium enriched with 10% FBS and then incubated at 37 °C for 3 h with 5% CO_2_ and 95% humidity [40]. Subsequently, Huvecs were infected in vitro with trypomastigotes at a ratio of 10:1 (parasites/cell) and incubated at 37 °C for 24 h with 5% CO_2_ in Dulbecco’s Modified Eagle Medium (DMEM, Sigma-Aldrich) enriched with 10% FBS. In vitro differentiation of amastigotes and Huvecs was performed after trypan blue staining [43].

### 2.12. Cytotoxicity Assay

For the cytotoxicity assay, Huvecs were used at a concentration of 1.4 × 10^5^ cells/mL. Cells were seeded in 24-well plates containing DMEM supplemented with 10% fetal bovine serum (FBS) and incubated at 37 °C with 5% CO_2_ in a humidified atmosphere for 72 h. After incubation, cells were exposed to different concentrations (50–300 μg/mL) of the actinomycete-derived extracellular metabolites. Cells treated with 1% DMSO and untreated cells were included as negative controls. Following treatment, cells were incubated for 24 h at 37 °C, and cytotoxicity was evaluated using the MTT colorimetric assay at 540 nm [44].

The median lethal concentration (LC_50_) was calculated with a 95% confidence interval. Extracellular metabolites were classified according to their cytotoxicity as follows: highly cytotoxic (LC_50_ < 10 μg/mL), cytotoxic (10 < LC_50_ < 100 μg/mL), moderately cytotoxic (100 < LC_50_ < 1000 μg/mL), and potentially non-cytotoxic (LC_50_ > 1000 μg/mL) [45].

In addition, the selectivity index (SI) of the extracellular metabolites was calculated as described, which indicates the relationship between the cytotoxic LC_50_ and the LC_50_ of the target parasite [46]. The extracellular metabolites were considered to be non-selective (SI < 2), moderately selective (2 < SI < 3), or selective (SI > 3) with respect to *T. cruzi* [47].

A higher selectivity index (SI) indicates greater activity of the extracellular metabolites against parasites compared to human cells, which helps to minimize the occurrence of adverse effects caused by them [48].

### 2.13. Fractionation of Extracellular Metabolites by Column Chromatography

Before fractionation, previous solubility tests were conducted using n-hexane, chloroform, ethyl acetate, and methanol (for fractionation steps, all reagents and materials were acquired from Sigma-Aldrich) [49]; however, hexane was excluded as a solvent in the fractionation assay. The chromatographic columns (22 × 300 mm) were packed with 20 g of silica gel 60 G (70–230 µm, 60–200 µm), and 500 mg of the ST-C43 extracellular metabolites and 500 mg of the S-C104 extracellular metabolites were separately loaded into the columns. Fractionation was performed with 20 mL of a solvent system from lower to higher polarity; first, both extracts were eluted with chloroform in different columns, followed by 20 mL of chloroform and ethyl acetate (9:1). The eluted fractions were labeled as ST-C43-CEA and S-C104-CEA. Afterwards, the proportion of ethyl acetate was gradually increased until only pure ethyl acetate was added to the columns. Then, these fractions were mixed with methanol in a 9:1 ratio, and the concentration of methanol was gradually increased until pure methanol was eluted through the column. These fractions were labeled as S-C43-EAM and S-C104-EAM. Supplementary Table S1 provides a detailed description of the gradient solvent systems used for the fractionation of extracellular metabolites. Each fraction was evaporated using a rotary evaporator. Thin-layer chromatography (TLC) was then performed to combine fractions with similar chromatographic profiles [50].

### 2.14. LC-MS/MS Analysis

The fraction with >10 SI (ST-C43-EAM-F2) was used for determination of its compound profile via LC-MS/MS analysis on a Thermo Fischer UHPLC system (Thermo Fisher Scientific, Emeryville, CA, USA) with an electron spray interference (ESI). The assay was performed as previously described [50]. ESI was used as a peak identifier in a positive ion mode. The product ion scans for each analyte were carried out by directly infusing 10 μL min^−1^ of individual standard solutions (1 mg L^−1^) using the built-in syringe pump. The infusion passed through a T-junction, where it was mixed with the blank column eluate at a flow rate of 200 μL min^−1^. The UNIFI 1.8 software (Waters Tm, Waters Corporation, Milford, MA, USA) and the Natural Products Atlas were used for analyzing the identified mass spectra [51,52,53].

### 2.15. Statistical Analysis

The inhibition results were analyzed with a dose–response regression test with a 95% confidence interval. A PROBIT test was carried out to calculate the IC_50_ (half-maximal inhibitory concentration) and LC_50_ (half-maximal lethal concentration) values, as well as the lower and upper limits. The post hoc Tukey’s honest significance test (HSD) was performed to determine any statistical differences between treatments. The analyses were performed using the SPSS software (Statistical Package for the Social Sciences, v 25.0, IBM Corp., Armonk, NY, USA). All assays were performed in triplicate, and data are presented as means of all replicates ± standard deviation (SD), and were previously checked for normality and homoscedasticity using the Shapiro–Wilk and Brown–Forsythe tests, respectively [54].

## 3. Results

### 3.1. Inhibitory Activity of Streptomyces Cell Cultures Against T. cruzi Epimastigotes

The number of *T. cruzi* epimastigotes decreased proportionally, according to the increased concentration of *Streptomyces*. In particular, at a concentration of 5 × 10^7^ cells/mL, ST-C43 and S-C104 inhibited 97.75% and 97.5% of protozoans, respectively (Table 1).

### 3.2. Antiparasitic/Inhibitory Activity of Purified Streptomyces Extracellular Metabolites Against Multiple T. cruzi Forms

All inhibition assays with extracellular metabolites from ST-C43 and S-C104 demonstrated no toxic, cytotoxic, or hemolytic activity, with an upper concentration of 300 µg/mL. For epimastigotes, ST-C43 and S-C104 strains demonstrated LC_50_ values of 100–110 µg/mL and 102–116 µg/mL for trypomastigotes; however, only ST-C43 showed strong activity against amastigote forms (Table 2, Supplementary Figure S1).

### 3.3. Trypanocidal, Toxic, and Hemolytic Activity of the Fractions

From the ethyl acetate–methanol extracellular metabolites, four fractions were obtained from the ST-C43 strain and three from the S-C104 strain. Trypanocidal activity assays revealed that fractions 1–4 from ST-C43-EAM exhibited LC_50_ values ranging from 105 to 116 µg/mL against the epimastigote stage of *T. cruzi*. These fractions were not toxic and not cytotoxic with respect to *A. salina* and Huvecs, with an upper concentration of 300 µg/mL from the fractions. They did not exhibit hemolytic activity on erythrocytes except for F1 and F3, which showed hemolytic activity at higher concentrations (245 and 256 µg/mL, respectively). However, S-C104 fractions showed no anti-*Trypanosoma*, toxic, or hemolytic activity (Table 3).

### 3.4. Identification of Chemical Compounds

**Figure 1:**
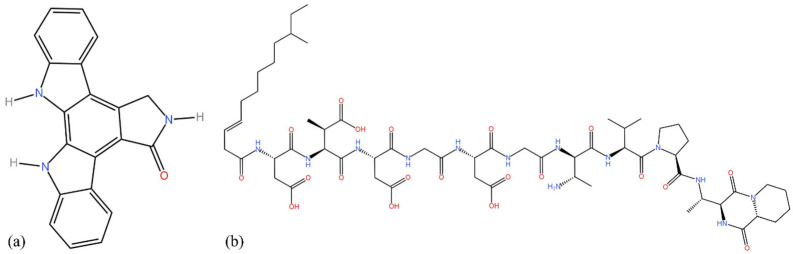
Chemical structures of (**a**) K-252-C-Aglycone and (**b**) amphomycin.

As only the fractions from ST-C43-EAM showed trypanocidal activity, these were selected for identification via LC-MS/MS. Two compounds were identified: K-252-C-Aglycone staurosporine (from the alkaloid family) and amphomycin (an antibiotic). The LC-MS/MS data for these compounds are given in Table 4, and their chemical structures are detailed in Figure 1.

## 4. Discussion

This study presents evidence regarding the potential use of actinomycete-derived extracellular metabolites as biocontrol agents against *Trypanosoma cruzi*—the etiological agent of Chagas disease (ChD)—with particular interest in the strain isolated from Nuevo León, Mexico [55]. Actinomycetes are well known as prolific producers of bioactive compounds and, among them, the genus *Streptomyces* has been extensively studied for its antimicrobial, antitumor, and anti-inflammatory properties [56,57]. Based on this, extracellular metabolites from the strains *Streptomyces thermocarboxydus* ST-C43 and *Streptomyces* sp. S-C104 were evaluated for their trypanocidal activities against *T. cruzi*.

Previous studies have demonstrated the effectiveness of *Streptomyces* species against various protozoa, including phenazine-carboxylic acid from *S. canus* against *T. gondii* and *P. falciparum* [16], orlistat isolated from *S. toxytricini* against *Giardia lamblia* [58], and other actinomycetes against *Entamoeba histolytica* [59]. In the case of hemoflagellate parasites, venturicidin A was shown to be highly active against *L. donovani* and *T. brucei i*, the latter being the causative agent of African trypanosomiasis [17]; however, reports on bioactive metabolites from *Streptomyces* species with activity against *T. cruzi* remain limited [18].

In this study, the extraction of extracellular compounds produced by *Streptomyces* in ISP2 medium after 15 days of incubation resulted in a yield of 0.04%. Although this yield is low, similar incubation times have been reported for species such as *S. fradiae* and *S. lomondensis*, with incubation periods ranging from 48 h to 21 days, depending on the strain and culture conditions [60,61,62]. To date, no data have been reported on the metabolite production of *S. thermocarboxydus*. However, efforts to optimize fermentation conditions—such as culture medium composition, aeration, and incubation time—have been shown to improve yields in other *Streptomyces* species [63]. This suggests that *S. thermocarboxydus* may require longer incubation times or specific fermentation conditions to increase the extract yield and production of bioactive compounds.

Regarding the antagonism tests of S-C104 and ST-C43 strains against *T. cruzi*, the actinomycete extracellular metabolites demonstrated strong inhibitory effects, with growth inhibition percentages ranging from 92.33% to 97.75%, respectively. Previous studies have shown that members of the genus *Streptomyces* can effectively inhibit the growth of pathogenic bacteria, achieving inhibition rates of up to 100% [64]. In the case of hemoflagellate protozoans such as *Leishmania* spp., antagonistic effects have been reported using concentrations of 1–2 × 10^8^ colony-forming units per milliliter (CFU/mL) of streptomyces [25]. However, no prior antagonistic studies against *T. cruzi* using similar actinomycete concentrations have been reported. Despite this, the high inhibition percentages observed in this study suggest that these Streptomyces may produce bioactive metabolites with known trypanocidal activity—such as avermectins—contributing to the observed mortality of *T. cruzi* cells [65].

Although *S. thermocarboxydus* belongs to the highly studied *Streptomyces* genus—widely recognized for its antiparasitic potential—few studies have specifically addressed its biological activities [11]. In our study, the trypanocidal activities of the extracellular metabolites revealed LC_50_ values of 100 µg/mL for the ST-C43 strain and 110 µg/mL for the S-C104 strain against the epimastigote forms of *T. cruzi*. These values are higher than those previously reported against *T. brucei*, with an LC_50_ of 16.6 µg/mL at the same developmental stage [66]. Similarly, LC_50_ values of 102 µg/mL for the ST-C43 strain and 116 µg/mL for the S-C104 strain were obtained against the trypomastigote forms, in contrast with earlier studies that reported values as low as 1.67 µg/mL [67]. These differences may be attributed to the culture medium used (ISP2), which could contain precursors—such as proteins—that influence metabolite production.

In relation to the bioassays with amastigotes, inhibition concentrations of 191 µg/mL were obtained, which contrasts with the previous studies [18] in which no activity was observed under similar culture conditions. Nonetheless, the LC_50_ values obtained in this study fall within the general range reported for *Streptomyces* extracellular metabolites against *T. brucei* (4–150 µg/mL) [68]. Moreover, both the ST-C43 and S-C104 extracellular metabolites showed no toxic activity in the *A. salina* assay, no hemolytic effect on erythrocytes, and no cytotoxicity toward Huvecs. These findings support the safety profile of the extracellular metabolites, as confirmed by their selectivity index (SI) > 10, thus indicating a low risk to human health [69].

Further fractionation of the ST-C43 extracellular metabolites (fractions ST-43-EAM-F1 to F4) retained antiparasitic activity with LC_50_ values ranging from 105 to 116 µg/mL, which aligns with prior findings [68]. However, fractions from S-C104 (S-C104-EAM-F1 to F3) failed to inhibit *T. cruzi*—possibly due to the absence of active compounds or the need for synergistic interactions for efficacy [70]. Additionally, ST-43-EAM-F1 and F3 exhibited hemolytic activity at concentrations of 245 and 256 µg/mL, respectively. While saponins were not detected—as previously implicated in hemolysis [44]—the activity may result from other bioactive metabolites that are harmful to human cells [70].

Consequently, subsequent assays excluded S-C104 and the hemolytic fractions ST-43-EAM-F1 and -F3 due to their lack of antiparasitic activity or safety concerns. This could be related to the absence of biosynthetic gene clusters responsible for producing antiparasitic compounds in S-C104 compared to ST-C43 [71]; this may be due to the presence of nitrate reductase, an enzyme that directly affects the metabolism of *T. cruzi,* similarly to benznidazole and nifurtimox [72]. Biochemical profiling of the extracellular metabolites identified the presence of carbohydrates, amino acids, phenols, alkaloids, coumarins, and sesquiterpene lactones—compounds known to play multiple biological roles, including biocontrol and antitumor activities [73,74,75]. The moderate LC_50_ values ranged from 100 to 191 µg/mL—a very low range of doses, when compared to benznidazole (5–7 mg/kg/day for adults) and nifurtimox (8–10 mg/kg/day) [72].

Notably, the ST-43-EAM-F2 fraction contained amphomycin, a compound previously reported as having antiparasitic activity against *Plasmodium falciparum* and *Toxoplasma gondii* [16] and recognized for its ability to inhibit peptidoglycan synthesis in Gram-positive bacteria [76]. K-252-C-Aglycone (staurosporine)—an alkaloid isolated from *Streptomyces* sp. strain 196 and *S. staurosporeus*—was also present, which is known to inhibit protein kinases [76] as well as possessing antibacterial [77] and antifungal properties [78,79]. These findings support previous observations that proteinaceous compounds from Streptomyces—especially those from *Streptomyces*—can exert antiparasitic effects [80], although they are not the only compounds implicated in activity against *Trypanosoma* spp. Staurosporine belongs to the alkaloid family, and has been described as a DNA synthesis inhibitor in eukaryotic cells [81]; meanwhile, amphomycin is a lipopeptide that inhibits the synthesis of dolichol-linked saccharides in eukaryotic cells, thus interfering with the glycosylation of glycoproteins [82].

In summary, this study demonstrates the potential of *S. thermocarboxydus* ST-C43 and *Streptomyces* sp. S-C104 as promising sources of antiparasitic compounds against *T. cruzi*. Although the activity observed was moderate compared to previous reports against *T. brucei*, the absence of toxicity in biological safety models coupled with the identification of bioactive metabolites (e.g., amphomycin and staurosporine) supports their potential use in biocontrol strategies. The results suggest that specific culture conditions and compound synergism may influence the resulting antiparasitic efficacy. Further investigations involving compound purification, mechanism of action studies, and genomic exploration of biosynthetic gene clusters are warranted to optimize the therapeutic potential of the considered Streptomyces-derived extracellular metabolites.

## 5. Conclusions

Extracellular metabolites from ST-C43 and S-C104 demonstrated inhibitory activities against *Trypanosoma cruzi*, with LC_50_ values ranging from 102 to 191 µg/mL. These extracellular metabolites were characterized as non-toxic, non-cytotoxic, and non-hemolytic, and showed favorable selectivity indices. Particularly, the ST-C43-EAM-F2 and ST-C43-EAM-F4 fractions derived from ST-C43 exhibited LC_50_ values between 115 and 124 µg/mL while retaining a safety profile rendering them suitable for potential therapeutic use. Chemical analyses revealed the presence of amphomycin and K-252-C-aglycone staurosporine—compounds previously reported for their antiparasitic properties. These findings underscore the potential of ST-C43-derived metabolites as promising candidates for the development of new treatments against ChD. Conversely, fractions from S-C104 did not exhibit significant trypanocidal activity. In conclusion, the bioactive compounds identified in *Streptomyces* extracellular metabolites—particularly from ST-C43—represent a valuable source for the development of novel antiparasitic agents. Nonetheless, further in vivo studies, including preclinical evaluations, are required to validate their efficacy and safety before advancing toward clinical application.

## Figures and Tables

**Table 1:** Inhibition percentages in the antagonism assay of ST-C43 and S-C104 cultures vs. epimastigote forms of *T. cruzi*.

Cell Concentration of *Streptomyces* (CFU/mL)	*T. cruzi* Epimastigotes Mortality (%) +/− SD	
	ST-C43	S-C104
1 × 10^7^	93.25 ± 0.07 ^a^	92.33 ± 2.35 ^a^
2 × 10^7^	94.25 ± 0.14 ^a^	93.33 ± 2.36 ^a^
3 × 10^7^	95.17 ± 1.01 ^a^	94.50 ± 1.47 ^a^
4 × 10^7^	96.42 ± 1.02 ^a^	96.00 ± 3.43 ^a^
5 × 10^7^	97.75 ± 1.89 ^b^	97.50 ± 2.17 ^b^

*Trypanosoma cruzi* at 1.2 × 10^6^ cells/mL. All assays were performed in triplicate. Inhibition percentages are the means ± SD, with significant (*p* < 0.05) differences indicated by different letters within the same column, as determined using the post hoc Tukey’s test.

**Table 2:** Evaluation of trypanocidal, toxic, cytotoxic, and hemolytic activities, as well as selectivity index of the extracellular metabolites of ST-C43 and S-C104 (LC_50_ in µg/mL, LL, and UL).

Activity	ST-C43	S-C104
Epimastigotes	100 (89–108)	110 (91–121)
Trypomastigotes	102 (91–107)	116 (97–129)
Amastigotes	198 (178–217)	>300
Toxicity	>1000 (not toxic)	>1000 (not toxic)
Cytotoxicity	>1000 (not cytotoxic)	>1000 (not cytotoxic)
Hemolysis	>1000 (not hemolytic)	>1000 (not hemolytic)
SI	>10	>10

LC_50_: lethal concentration 50; LL: lower limit; UL: upper limit; toxicity evaluated in *Artemia salina*, cytotoxicity evaluated in Huvecs, hemolysis evaluated on human red cells; SI: selectivity index.

**Table 3:** Evaluation of trypanocidal, toxic, and hemolytic activities of the fractions of ST-C43 and S-C104 (LC_50_ in µg/mL, LL, and UL).

Fraction	Activity			
	Anti-*Trypanosoma*	Toxic	Hemolytic	SI
ST-C43-EAM-F1	105 (96–108)	>1000 *	245 (227–266)	>10
ST-C43-EAM-F2	115 (102–122)	>1000 *	>1000 **	>10
ST-C43-EAM-F3	107 (100–116)	>1000 *	256 (240–271)	>10
ST-C43-EAM-F4	116 (110–128)	>1000 *	>1000 **	>10
S-C104-EAM-F1	>1000 (no activity)	>1000 *	>1000 **	N.A.
S-C104-EAM-F2	>1000 (no activity)	>1000 *	>1000 **	N.A.
S-C104-EAM-F3	>1000 (no activity)	>1000 *	>1000 **	N.A.

LC_50_: lethal concentration 50; LL: lower limit; UL: upper limit; *: non-toxic in *A. salina*; **: non-hemolytic; N.A.: not applicable. SI: selectivity index.

**Table 4:** List of compounds identified from ST-C43-EAM-F2 via LC-MS/MS.

Unit	Retention Time (min)	Molecular Weight	*m*/*z* Value
Amphomycin	12.28	1290.4	1318.6900
K-252-C-Aglycone	10.54	311.4	334.0501

## Data Availability

The datasets generated or analyzed during the present study are available on request from the corresponding author Z.J.M-G.

## Supplementary Materials

The following supporting information can be downloaded at: https://www.mdpi.com/article/10.3390/pathogens14070638/s1: Supplementary Figure S1. Plots of LC_50_ to calculate the antiparasitic activity of the extracellular metabolites of (a) ST-C43 and (b) S-C104 against epimastigotes, trypomastigote and amastigote forms of *Trypanosoma cruzi* forms. Supplementary Table S1. Gradient solvent system used for the fractionation of extracellular metabolites of ST-C43 and S-C104.
