# Supplementary material for: Inhibitory Activity of Compounds Obtained from Streptomyces Against Trypanosoma cruzi
Source: Pathogens. 2025 Jun 26;14(7):638. doi: 10.3390/pathogens14070638 (PMC12300863; doi:10.3390/pathogens14070638)
Supplement: Supplementary file 1 [file pathogens-14-00638-s001.zip › pathogens-3687448-supplementary.pdf]

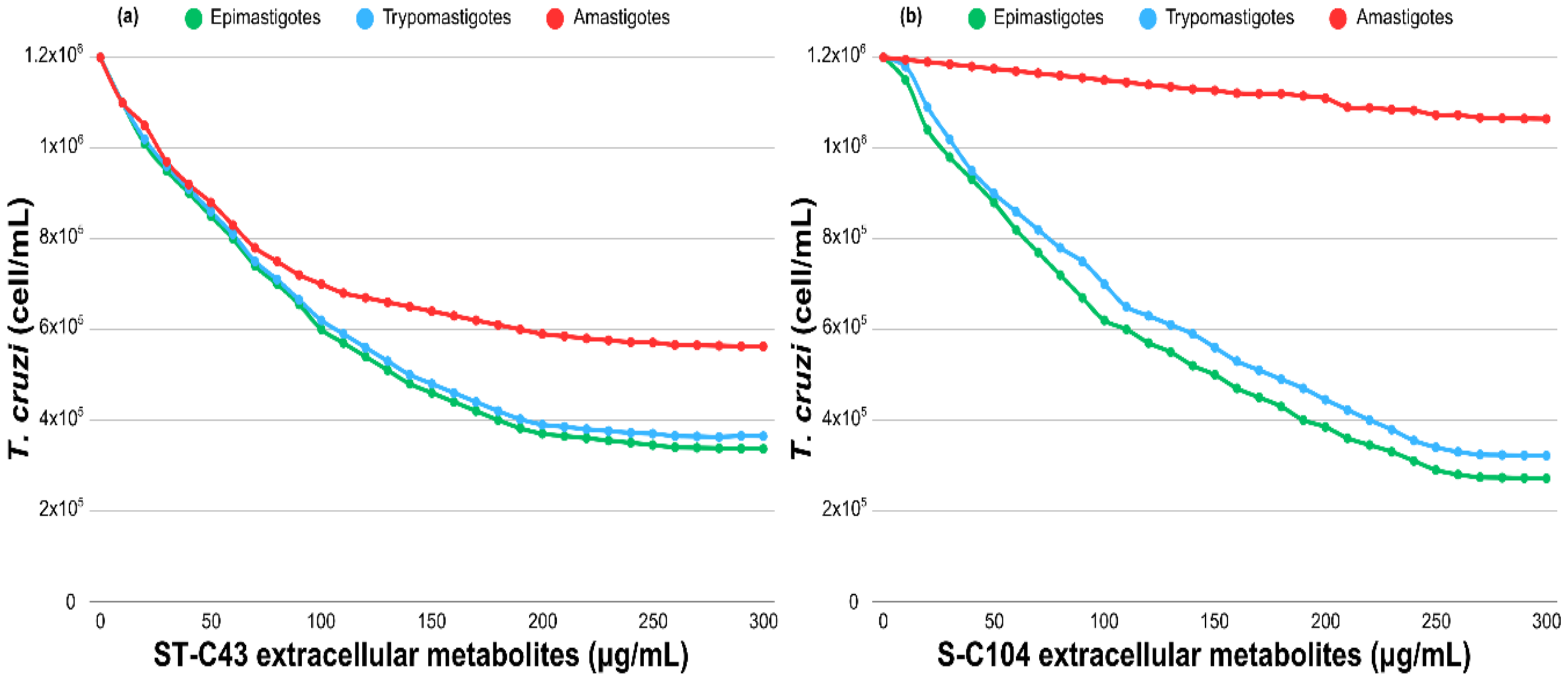

Supplementary Figure S1. Plots of LC_{50} to calculate the antiparasitic activity of the extracellular metabolites of (a) ST-C43 and (b) S-C104 against epimastigotes, trypomastigote and amastigote forms of *Trypanosoma cruzi* forms.

Supplementary Table S1. Gradient solvent system used for fractionation of extracellular metabolites of ST-C43 and S-C104.

Gradient
Chloroform 100%
Chloroform -Ethyl acetate 9:1
Chloroform - Ethyl acetate 8:2
Chloroform - Ethyl acetate 7:3
Chloroform - Ethyl acetate 6:4
Chloroform - Ethyl acetate 5:5
Chloroform - Ethyl acetate 4:6
Chloroform - Ethyl acetate 3:7
Chloroform - Ethyl acetate 2:8
Chloroform - Ethyl acetate 1:9
Ethyl acetate 100%
Ethyl acetate -Methanol 9:1
Ethyl acetate -Methanol 8:2
Ethyl acetate -Methanol 7:3
Ethyl acetate -Methanol 6:4
Ethyl acetate -Methanol 5:5
Ethyl acetate -Methanol 4:6
Ethyl acetate -Methanol 3:7
Ethyl acetate -Methanol 2:8
Ethyl acetate -Methanol 1:9
Methanol 100%
